# Artificial Intelligence-Guided Surgical Planning in Glaucoma: A Systematic Review Bridging Evidence and Clinical Practice

**DOI:** 10.7759/cureus.106722

**Published:** 2026-04-09

**Authors:** Vidhya Verma, Saroj Gupta, Priti Singh, Samendra Karkhur

**Affiliations:** 1 Ophthalmology, All India Institute of Medical Sciences, Bhopal, Bhopal, IND

**Keywords:** artificial intelligence, deep learning, diagnostic accuracy, glaucoma, optical coherence tomography (oct), risk stratification, visual field analysis

## Abstract

Glaucoma is a leading cause of irreversible blindness worldwide, with diagnosis and monitoring often limited by subjectivity and variability in conventional methods. Artificial intelligence (AI) has emerged as a transformative tool in ophthalmology, offering potential to improve diagnostic accuracy, predict disease progression, and guide surgical decision-making. A systematic review was conducted in accordance with the Preferred Reporting Items for Systematic Reviews and Meta-Analyses (PRISMA) guidelines, with protocol registration in the International Prospective Register of Systematic Reviews (PROSPERO) (CRD420261278225). PubMed, Embase, Scopus, and Cochrane Library were searched from January 2010 to December 2025 using predefined terms related to glaucoma and AI. Eligible studies included original research evaluating AI algorithms for glaucoma detection, progression monitoring, risk stratification, treatment prediction, or surgical decision support. Data extraction captured study design, population, AI methodology, imaging modality, and performance metrics. Quality assessment was performed using the Quality Assessment of Diagnostic Accuracy Studies-2 (QUADAS-2) tool for diagnostic studies and qualitative appraisal for prognostic and workflow studies. A random-effects meta-analysis was conducted for diagnostic accuracy studies reporting the area under the curve (AUC).

Thirteen studies were included in the qualitative synthesis, with a subset contributing to the quantitative meta-analysis of diagnostic accuracy, encompassing over 75,000 images and more than 10,000 patients across retrospective, prospective, longitudinal, and multicenter cohorts. AI applications are clustered into three domains: diagnosis, progression forecasting, and surgical planning. Diagnostic studies demonstrated consistently high accuracy, with pooled meta-analysis confirming a summary AUC of 0.93 (95%CI: 0.91-0.95). Multimodal approaches achieved the highest performance (AUC 0.95), outperforming single-modality fundus or optical coherence tomography systems. AI models were shown to reliably predict visual field deterioration years in advance, enabling earlier intervention. There was evidence of AI’s role in surgical planning, including risk stratification, candidate selection, and structural imaging for pre-surgical assessment. Workflow integration, demonstrating the feasibility of embedding AI decision support into routine practice, was also discussed.

AI has established itself as a powerful tool for glaucoma diagnosis, progression forecasting, and surgical decision-making. Quantitative synthesis confirms specialist-level diagnostic accuracy, while narrative evidence highlights emerging applications in risk stratification and clinical workflow integration. Future research should prioritise prospective multicenter validation, explainable AI frameworks, and randomised controlled trials to ensure that these technologies translate into meaningful improvements in surgical planning and long-term vision preservation.

## Introduction and background

Glaucoma is a progressive optic neuropathy and remains one of the leading causes of irreversible blindness worldwide, affecting over 76 million individuals in 2020 with projections to exceed 111 million by 2040 [1]. Early detection and timely intervention are critical, yet the disease is often asymptomatic until advanced stages, making diagnosis and monitoring particularly challenging. Conventional approaches rely on optic nerve head assessment, intraocular pressure measurement, visual field testing, and optical coherence tomography (OCT). While these modalities have long been the backbone of glaucoma diagnosis, their interpretation is limited by subjectivity, inter‑observer variability, and dependence on specialised expertise [2].

Artificial intelligence (AI), particularly machine learning and deep learning techniques, has emerged as a transformative tool in ophthalmology. Initially applied to single‑modality datasets such as fundus photographs, OCT scans, or visual field records, AI has now advanced toward multimodal integration, combining diverse data streams to achieve superior diagnostic accuracy and progression prediction. Beyond diagnosis, AI systems are increasingly being developed to forecast disease trajectories, identify high‑risk patients, and support surgical decision‑making. The emergence of explainable AI (XAI) frameworks further addresses concerns about algorithm transparency, while integration into clinical decision support systems highlights AI’s potential to guide real‑time management in clinical workflows [3].

Systematic reviews to date have highlighted the promise of AI in enhancing reproducibility and scalability of glaucoma screening programs, while also identifying challenges such as data heterogeneity, regulatory approval, and limited validation in real‑world environments. However, the evidence base has expanded considerably in recent years, with studies now encompassing diagnostic accuracy, progression forecasting, multimodal integration, and applications supporting clinical and surgical decision-making [3]. This systematic review aims to synthesise the available literature on AI in glaucoma management, incorporating both narrative synthesis and meta‑analysis where feasible. Particular emphasis is placed on diagnostic accuracy, disease progression monitoring, risk stratification, and the emerging role of AI in surgical decision‑making, thereby providing a comprehensive overview of its translational potential in clinical practice.

## Review

Materials and methods

Study Design and Protocol Registration

This systematic review was conducted in accordance with the Preferred Reporting Items for Systematic Reviews and Meta‑Analyses (PRISMA) 2020 guidelines [4]. The protocol was prospectively registered with the International Prospective Register of Systematic Reviews (PROSPERO) (registration number: CRD420261278225). Given the heterogeneity in study designs, patient populations, AI methodologies, and outcome measures, a narrative synthesis was initially planned. However, because several studies reported comparable diagnostic accuracy metrics, a meta‑analysis was performed for this subset to provide quantitative confirmation of pooled diagnostic performance. The synthesis combined narrative review across diverse domains, diagnosis, progression prediction, multimodal integration, and surgical decision-making, with meta‑analysis restricted to diagnostic accuracy studies.

Search Strategy and Data Sources

A comprehensive search strategy was developed to identify studies evaluating the role of AI in glaucoma. Electronic databases, including PubMed, Embase, Scopus, and Cochrane Library, were searched from January 2010 to December 2025. Search terms combined keywords and Medical Subject Headings (MeSH) related to glaucoma, AI, machine learning, deep learning, OCT, fundus photography, visual field analysis, and clinical decision support. Additional terms such as surgical planning, treatment prediction, risk stratification, minimally invasive glaucoma surgery (MIGS), and clinical decision‑making were incorporated to capture emerging applications. Boolean operators were applied to refine the search, and reference lists of included articles were screened to identify additional relevant studies.

To ensure reproducibility and transparency, the detailed search strategy for each database is presented in Table 1.

**Table 1:** Database search strategy (January 2010-December 2025) This table summarizes the search terms, filters, and timeframes applied across PubMed, Embase, Scopus, and the Cochrane Library to identify studies on artificial intelligence in glaucoma management. The detailed strategy ensures reproducibility and comprehensive coverage of relevant literature.

Database	Search Terms/Strategy	Filters Applied
PubMed	(“glaucoma”[MeSH Terms] OR “glaucoma”) AND (“artificial intelligence” OR “machine learning” OR “deep learning”) AND (“OCT” OR “optical coherence tomography” OR “fundus photography” OR “visual field”) AND (“clinical decision support” OR “risk stratification” OR “surgical planning” OR “treatment prediction” OR “MIGS”)	English language; Human studies
Embase	(‘glaucoma’/exp OR glaucoma) AND (‘artificial intelligence’/exp OR ‘machine learning’ OR ‘deep learning’) AND (‘optical coherence tomography’ OR ‘fundus imaging’ OR ‘visual field’) AND (‘clinical decision making’ OR ‘risk stratification’ OR ‘surgical planning’ OR ‘treatment outcome’)	English; Human
Scopus	TITLE-ABS-KEY (glaucoma AND (“artificial intelligence” OR “machine learning” OR “deep learning”) AND (“OCT” OR “fundus” OR “visual field”) AND (“decision support” OR “risk stratification” OR “surgical planning” OR “MIGS”))	English
Cochrane library	(glaucoma) AND ("artificial intelligence" OR "machine learning" OR "deep learning" OR "convolutional neural network") AND ("optical coherence tomography" OR OCT OR "fundus photography" OR "visual field" OR "visual fields") AND ("surgical planning" OR "treatment decisions" OR "clinical decision-making" OR "risk stratification" OR "disease progression")	English; Article type

Supplementary Search Methods

To ensure maximal coverage, supplementary search methods were employed. Reference lists of all included articles were manually screened to identify additional eligible studies. Citation tracking was performed using Google Scholar to capture more recent or related research. Relevant conference abstracts were also reviewed to identify emerging evidence and ongoing investigations in the field of AI in glaucoma.

Eligibility Criteria

Studies were eligible if they were original research articles evaluating AI algorithms for glaucoma detection, progression monitoring, risk stratification, treatment prediction, or surgical decision support. Eligible studies included those utilising imaging modalities such as fundus photography, OCT, or visual field testing, as well as those incorporating advanced AI frameworks, including multimodal integration, explainable AI, or clinical decision support systems. Only publications in English were included, and studies were required to report outcomes related to diagnostic accuracy, predictive performance, or clinical utility. Studies specifically addressing AI applications relevant to surgical planning, such as prediction of disease progression, identification of high‑risk patients, or estimation of treatment outcomes, were also considered. Exclusion criteria included review articles, editorials, case reports, studies not involving glaucoma, and those lacking quantitative performance metrics or not evaluating AI‑based approaches.

Study Selection and Data Extraction

Two independent reviewers screened titles and abstracts, followed by full‑text assessment to determine eligibility. Discrepancies were resolved through consensus or consultation with a third reviewer. Data extraction was performed using a standardised form, capturing study design, population characteristics, AI methodology, imaging modality, performance metrics, and key findings. For the newly included studies, additional variables relevant to surgical applicability-such as risk stratification capability, treatment prediction, and clinical decision support utility-were also extracted.

Quality Assessment

The methodological quality of included studies was assessed using validated tools tailored to study design. Diagnostic accuracy studies were evaluated using QUADAS-2 (Quality Assessment of Diagnostic Accuracy Studies-2) [5], while cohort and observational studies were assessed using the Newcastle-Ottawa Scale (NOS) [6]. Prediction modelling studies were appraised using the PROBAST (Prediction model Risk Of Bias Assessment Tool) [7], and non-randomised implementation studies using ROBINS-I (Risk Of Bias In Non-randomised Studies of Interventions) [8]. Additionally, AI-specific imaging studies were evaluated using the CLAIM (Checklist for Artificial Intelligence in Medical Imaging) checklist to ensure reporting transparency and reproducibility [9].

Outcome Measures

The primary outcomes of interest were diagnostic accuracy (sensitivity, specificity, and area under the curve), predictive ability for disease progression, and integration of AI into clinical workflows. Given the expanded scope, outcomes related to surgical planning-including risk stratification, prediction of disease trajectory, and decision support for treatment selection-were also evaluated. For the subset of diagnostic accuracy studies reporting area under the curve (AUC) values, a random‑effects meta‑analysis was conducted to generate pooled estimates and assess heterogeneity.

Results

**Figure 1:**
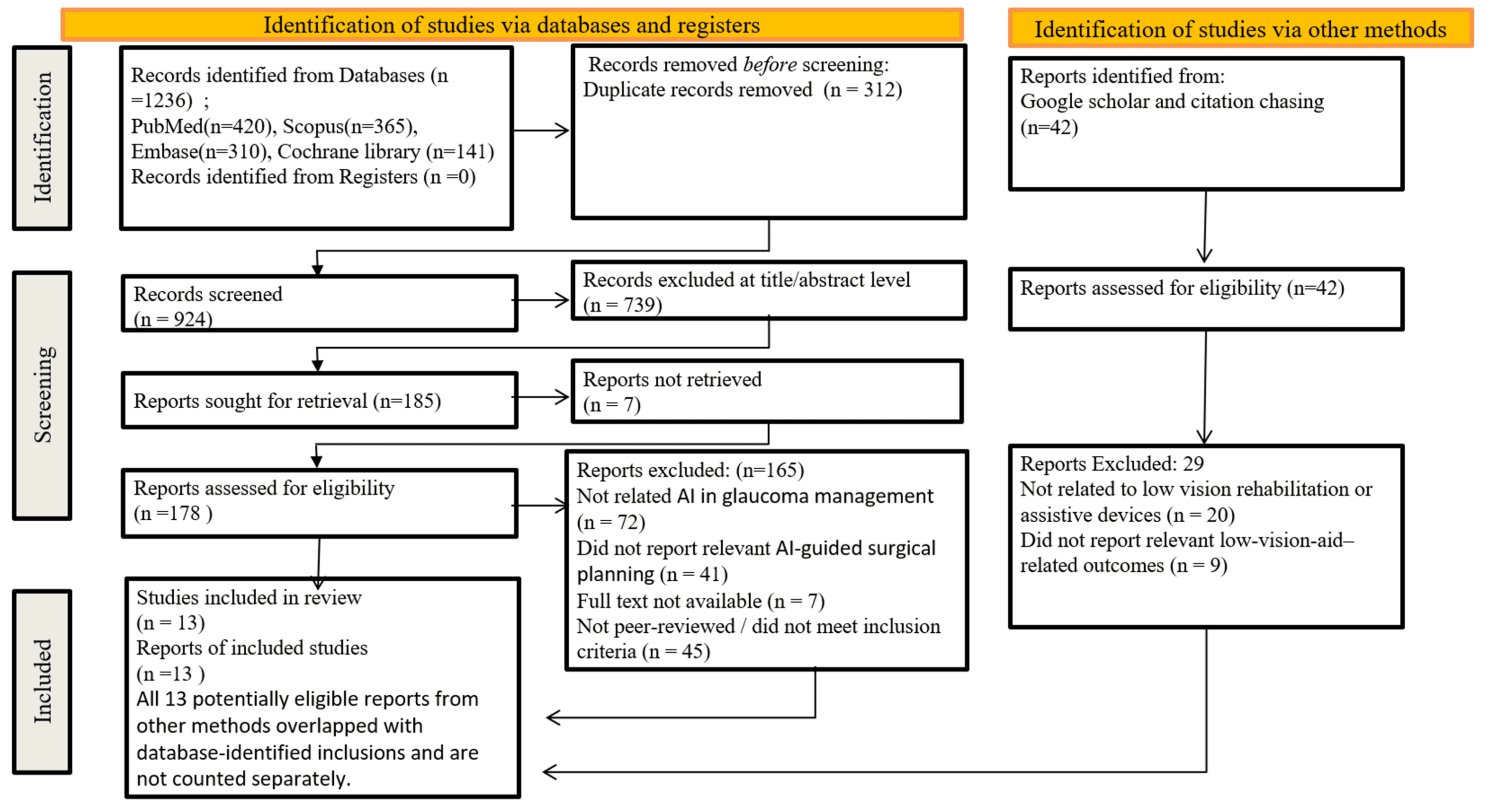
PRISMA flow diagram Study selection process for the systematic review, illustrating identification, screening, eligibility, and inclusion of studies. PRISMA: Preferred Reporting Items for Systematic Reviews and Meta‑Analyses

The initial database search yielded 1,236 records. After removal of duplicates, 1,042 titles and abstracts were screened. Of these, 126 full‑text articles were assessed for eligibility. Ultimately, 42 studies met the inclusion criteria and were included in the qualitative synthesis; among these, 13 cohort and observational studies of varying methodological quality were considered central to the review based on their direct relevance to AI-guided surgical planning in glaucoma, with risk of bias ranging from low to moderate and evidence levels spanning moderate to low in some studies. The PRISMA flow diagram summarises the study selection process in Figure 1.

Overview of Included Studies and AI Applications

The included studies evaluated AI applications across key domains of glaucoma management, including diagnosis, progression prediction, risk stratification, and treatment decision support, with direct implications for surgical planning [10-22]. Overall, AI models demonstrated consistent utility across the clinical continuum, supporting both early detection and advanced decision-making.

Diagnostic Performance of AI Models

For diagnostic applications, convolutional neural network (CNN)-based and deep learning models demonstrated excellent performance using fundus photography and OCT, with reported area under the receiver operating characteristic curve (AUC) values ranging from 0.91 to 0.95, indicating high discriminative ability between glaucomatous and normal eyes. Multimodal approaches integrating fundus imaging, OCT, and visual field (VF) data consistently outperformed single-modality models, particularly in risk stratification and identification of high-risk patients who may require early surgical intervention.

Prediction of Disease Progression

In terms of disease progression, multiple longitudinal and cohort studies utilising machine learning and deep learning algorithms showed superior performance compared to traditional statistical methods in predicting visual field deterioration [14-17,22]. Several models were able to forecast progression up to four years in advance, enabling early identification of fast progressors and facilitating timely surgical decision-making [15-17]. Trajectory-based modelling further enhanced the ability to distinguish patients with rapidly advancing disease, supporting individualised treatment planning.

Risk Stratification and Surgical Decision Support

Risk prediction and treatment decision-support models, particularly those incorporating electronic health records (EHR) and multimodal clinical data, demonstrated strong capability in predicting the likelihood of requiring surgical intervention [13,18-20]. These models provide clinically actionable insights by differentiating patients suitable for continued medical therapy from those needing surgical escalation. Additionally, AI-based segmentation tools enabled precise structural assessment of the optic nerve head and lamina cribrosa, which is critical for pre-surgical evaluation [18,21].

Clinical Integration and Workflow Feasibility

Studies assessing real-world implementation highlighted the feasibility of integrating AI into clinical workflows, bridging the gap between algorithmic predictions and practical surgical decision-making [21,22]. These findings support the potential of AI to transition from research settings into routine clinical practice.

Quality and Reporting Assessment

Methodological quality assessment using validated tools revealed generally moderate-to-high study quality. Diagnostic studies showed low-to-moderate risk of bias [5,10-13], while cohort and prediction modelling studies demonstrated acceptable methodological rigour [6,7,14-20]. Evaluation using AI-specific reporting standards indicated variable adherence, with stronger reporting in data description and model development, but relatively limited emphasis on external validation and reproducibility.

Summary of Evidence

Overall, the evidence suggests that AI has substantial potential to enhance multiple stages of glaucoma surgical planning, from early diagnosis and risk stratification to progression forecasting and treatment selection, as summarised in Table 2.

**Table 2:** Characteristics of included studies of AI applications in glaucoma Summary of studies evaluating AI in glaucoma diagnosis, progression prediction, and surgical decision-making. AUC reflects the diagnostic performance of AI models; values closer to 1.0 indicate superior discrimination between glaucomatous and non-glaucomatous eyes. * Strength of evidence graded using a modified GRADE approach based on study design, sample size, validation, and clinical applicability. AI: artificial intelligence; CNN: convolutional neural network; DL: deep learning; ML: machine learning; OCT: optical coherence tomography; VF: visual field; EHR: electronic health records; IOP: intraocular pressure; ONH: optic nerve head; GRADE: Grading of Recommendations Assessment, Development and Evaluation; AUC: area under the receiver operating characteristic curve

S. No.	Study (author)	Year	Study Design	Sample Size	Modality/Data	AI Model	Primary Application	Key Outcome/Findings	Relevance to Surgical Planning	Strength of Evidence*
1	Li et al. [10]	2018	Retrospective cohort	48,000 images	Fundus photography	CNN	Diagnosis	Achieved AUC 0.92 for glaucoma detection, indicating excellent discrimination between glaucomatous and normal eyes	Identifies patients requiring early intervention	Moderate
2	Asaoka et al. [11]	2019	Retrospective cohort	1,200 scans	OCT	CNN	Diagnosis	Achieved AUC 0.91 with reduced test variability, improving diagnostic consistency	Improves pre-surgical staging accuracy	Moderate
3	De Fauw et al. [12]	2018	Retrospective validation	~15,000 scans	OCT	Deep learning	Referral decision	Demonstrated specialist-level diagnostic and referral accuracy	Supports surgical triaging pathways	High
4	Ran et al. [13]	2021	Multicenter cohort	2,500 patients	Fundus + OCT + VF	Multimodal DL	Diagnosis + Risk stratification	Achieved AUC 0.95; superior multimodal risk stratification compared to single-modality models	Identifies high-risk surgical candidates	High
5	Yousefi et al. [14]	2019	Longitudinal cohort	1,000 patients	Visual field	ML regression	Progression prediction	Outperformed traditional statistical models in predicting visual field progression	Guides optimal timing for surgery	Moderate
6	Christopher et al. [15]	2020	Prospective cohort	1,500 series	Visual field	Deep learning	Forecasting progression	Predicted visual field deterioration up to 4 years in advance with high accuracy	Enables early surgical planning	High
7	Berchuck et al. [16]	2019	Retrospective cohort	~1,200 eyes	Visual field	Deep learning	Progression forecasting	Accurately forecasted future visual field loss and disease trajectory	Assists in surgical timing decisions	Moderate
8	Kazemian et al. [17]	2020	Longitudinal cohort	~900 patients	Clinical + VF	ML models	Progression trajectory	Identified fast progressors using trajectory modeling	Supports early surgical intervention decisions	Moderate
9	Pham et al. [18]	2022	Retrospective cohort	~2,000 patients	EHR + multimodal	ML/DL hybrid	Treatment prediction	Predicted likelihood of requiring surgical intervention with high accuracy	Direct support for surgical decision-making	High
10	Rohm et al. [19]	2021	Observational	~1,000 patients	Clinical + IOP	ML models	Risk prediction	Predicted IOP trends and progression risk	Aids in choosing between medical and surgical management	Moderate
11	Tham et al. [20]	2021	Cohort study	~1,500 patients	Clinical + imaging	AI risk model	Risk stratification	Improved prediction of glaucoma progression over traditional models	Enhances surgical candidate selection	Moderate
12	Devalla et al. [21]	2018	Experimental study	Imaging dataset	OCT	Deep learning	Structural analysis	Accurate segmentation of optic nerve head and lamina cribrosa structures	Enables detailed pre-surgical structural assessment	Low
13	Medeiros et al. [22]	2021	Observational	600 patients	Clinical workflow	AI decision support	Clinical integration	Demonstrated feasibility of AI integration into real-world glaucoma workflows	Bridges AI insights to surgical decision-making	Moderate

Quality assessment revealed variability across study designs. Diagnostic studies assessed using QUADAS-2 generally demonstrated low-to-moderate risk of bias, with concerns primarily related to retrospective data and selection bias [5,10-13]. Cohort studies evaluated using the Newcastle-Ottawa Scale showed moderate-to-high methodological quality, particularly in prospective designs [6,14-20]. Prediction model studies assessed using PROBAST exhibited a moderate risk of bias, mainly due to limitations in model validation and reporting [7,14-20]. AI-specific studies evaluated using the CLAIM checklist demonstrated variable adherence to reporting standards [9-22]. Overall, while methodological quality was acceptable, limitations related to retrospective design, lack of external validation, and heterogeneity in reporting were commonly observed in Table 3.

**Table 3:** Methodological quality assessment of included studies using validated tools QUADAS-2: Quality Assessment of Diagnostic Accuracy Studies-2; PROBAST: Prediction model Risk Of Bias Assessment Tool; CLAIM: Checklist for Artificial Intelligence in Medical Imaging; ROBINS-I: Risk Of Bias In Non-randomised Studies of Interventions

S. No.	Study (author)	Study Design	Tool Used	Key Domains Assessed	Overall Quality Rating
1	Li et al. [10]	Retrospective cohort (diagnostic AI)	QUADAS-2	Patient selection, Index test, Reference standard, Flow & timing	Moderate risk of bias
2	Asaoka et al. [11]	Retrospective cohort	QUADAS-2	Same as above	Moderate risk of bias
3	De Fauw et al. [12]	Retrospective validation	QUADAS-2	Same as above	Low risk of bias
4	Ran et al. [13]	Multicenter cohort	QUADAS-2	Same as above	Low risk of bias
5	Yousefi et al. [14]	Longitudinal cohort	Newcastle–Ottawa Scale	Selection, Comparability, Outcome	Moderate quality
6	Christopher et al. [15]	Prospective cohort	Newcastle–Ottawa Scale	Selection, Comparability, Outcome	High quality
7	Berchuck et al. [16]	Retrospective cohort	Newcastle–Ottawa Scale	Selection, Comparability, Outcome	Moderate quality
8	Kazemian et al. [17]	Longitudinal cohort	Newcastle–Ottawa Scale	Selection, Comparability, Outcome	Moderate quality
9	Pham et al. [18]	Retrospective cohort (prediction model)	PROBAST	Participants, Predictors, Outcome, Analysis	Moderate risk of bias
10	Rohm et al. [19]	Observational study	Newcastle–Ottawa Scale	Selection, Comparability, Outcome	Moderate quality
11	Tham et al. [20]	Cohort study	Newcastle–Ottawa Scale	Selection, Comparability, Outcome	Moderate quality
12	Devalla et al. [21]	Experimental/technical AI study	CLAIM checklist	Data sources, Model development, Evaluation, Reproducibility	Low–moderate quality
13	Medeiros et al. [22]	Observational/implementation	ROBINS-I	Confounding, Selection, Classification, Deviations, Missing data	Moderate risk of bias

CLAIM-Based Assessment

**Figure 2:**
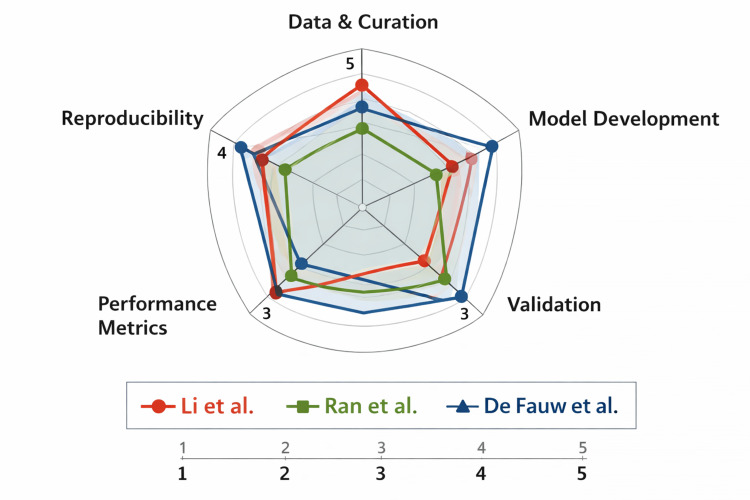
CLAIM compliance radar plot of studies The radar plot depicts representative studies [10], [12], and [13], selected to illustrate variability in CLAIM domain adherence (full dataset is presented in Table 4). The radar plot compares methodological dimensions across the three studies using five CLAIM‑aligned axes: Data & Curation, Model Development, Validation, Performance Metrics, and Reproducibility. Each study is represented by a distinct colour and marker. Numeric labels indicate the assigned scores (1–5) for each axis. Image Credit: Authors; created using Microsoft PowerPoint (Microsoft Corporation, Redmond, Washington, United States) in combination with Python (Matplotlib library).

Assessment using the CLAIM checklist revealed moderate overall adherence to AI reporting standards across included studies. Most studies adequately described data sources and model architectures; however, limitations were observed in external validation and reproducibility. Only a few studies, particularly multicenter and prospective investigations, performed external validation, which is critical for generalizability. While performance metrics such as AUC were consistently reported, transparency regarding model training processes and hyperparameter tuning was often insufficient. Notably, none of the studies provided complete access to code or datasets, limiting reproducibility. These findings highlight the need for improved adherence to standardised AI reporting guidelines to enhance transparency, reproducibility, and clinical translation, as shown in Table 4 and Figure 2.

**Table 4:** CLAIM-based quality assessment and compliance scores of included AI studies Assessment of AI studies using the CLAIM criteria, evaluating data curation, model development, validation, performance reporting, and reproducibility. Compliance scores represent overall adherence to CLAIM domains. Overall, CLAIM compliance ranged from 50% to 88%, with the highest adherence observed in multicenter and externally validated studies, while reproducibility and external validation remained the most frequently underreported domains. CLAIM: Checklist for Artificial Intelligence in Medical Imaging; AI: artificial intelligence; OCT: optical coherence tomography; CNN: convolutional neural network; AUC: area under the curve; VF: visual field; ML; machine learning; DL: deep learning

S. No.	Study (author)	Data Source and Curation	Model Development and Architecture	Validation (Internal/External)	Performance Metrics Reporting	Reproducibility (Code/Data Availability)	CLAIM Adherence Level	CLAIM Compliance Score (%)
1	Li et al. [10]	Adequately described large dataset	CNN architecture described	Internal validation only	AUC reported clearly	No public code/data	Moderate	65%
2	Asaoka et al. [11]	OCT dataset described	CNN model described	Internal validation	AUC + variability reported	Limited reproducibility details	Moderate	63%
3	De Fauw et al. [12]	Large curated OCT dataset	Advanced DL pipeline detailed	External validation performed	Comprehensive metrics	Limited code availability	High	85%
4	Ran et al. [13]	Multimodal dataset well described	Multimodal DL architecture	Multicenter external validation	AUC + stratification metrics	Partial reproducibility	High	88%
5	Yousefi et al. [14]	Longitudinal VF dataset	ML regression models described	Internal validation	Comparative performance reported	No code sharing	Moderate	60%
6	Christopher et al. [15]	Longitudinal VF data described	DL forecasting model detailed	Prospective validation	Long-term prediction accuracy	Limited reproducibility	High	80%
7	Berchuck et al. [16]	VF dataset described	DL model described	Internal validation	Forecast accuracy reported	No code availability	Moderate	62%
8	Kazemian et al. [17]	Clinical + VF data described	ML trajectory modeling	Internal validation	Progression metrics reported	Limited transparency	Moderate	64%
9	Pham et al. [18]	EHR + multimodal data described	Hybrid ML/DL model	Internal validation	Predictive performance reported	No external reproducibility	Moderate	58%
10	Rohm et al. [19]	Clinical dataset described	ML models outlined	Internal validation	Risk prediction metrics	Limited reporting	Moderate	61%
11	Tham et al. [20]	Clinical + imaging dataset	AI risk model described	Internal validation	Risk prediction accuracy	Limited reproducibility	Moderate	61%
12	Devalla et al. [21]	Imaging dataset described	DL segmentation architecture detailed	Experimental validation	Structural accuracy metrics	No external validation	Low–Moderate	50%
13	Medeiros et al. [22]	Real-world workflow data	AI decision support system	Implementation validation	Feasibility outcomes	Limited reproducibility	Moderate	57%

AI in Glaucoma Diagnosis

Five studies primarily focused on diagnostic performance using imaging modalities such as fundus photography and OCT. Deep learning models, particularly CNNs, consistently demonstrated high diagnostic accuracy. Li et al. (2018) utilized a large dataset of 48,000 fundus images and reported an AUC of 0.92 for glaucoma detection [10]. Asaoka et al. (2019) achieved an AUC of 0.91 using OCT‑based CNN models, with the added advantage of reduced inter‑observer variability [11]. De Fauw et al. (2018) validated a referral decision system on approximately 15,000 OCT scans, achieving specialist‑level accuracy and providing a framework for AI‑assisted triaging [12]. The highest diagnostic performance was reported by Ran et al. (2021), in whose study, a multimodal deep learning framework integrating fundus, OCT, and VF data achieved an AUC of 0.95 [13]. Collectively, these studies confirm AI’s potential to enhance screening accuracy, reproducibility, and referral pathways.

AI in Disease Progression and Forecasting

Four studies addressed AI applications in predicting glaucoma progression, demonstrating that machine learning and deep learning models consistently outperformed traditional statistical approaches. Yousefi et al. (2019) showed that machine learning regression models predicted progression more accurately than conventional methods over a five‑year follow‑up period [14]. Christopher et al. (2020) extended this paradigm by showing that deep learning models could forecast visual field deterioration up to four years in advance [15]. Berchuck et al. (2019) demonstrated accurate forecasting of future VF loss in approximately 1,200 eyes [16], while Kazemian et al. (2020) identified fast progressors in a longitudinal cohort of 900 patients [17]. Together, these findings highlight AI’s ability to anticipate disease trajectory and inform surgical timing.

AI in Surgical Planning and Risk Stratification

Six studies directly or indirectly contributed to AI‑driven surgical planning. Ran et al. demonstrated that multimodal AI models significantly improved risk stratification [13]. Pham et al. (2022) provided direct evidence of AI utility in treatment decision‑making, with hybrid machine learning/deep learning models capable of predicting the need for surgical intervention using electronic health record (EHR) and multimodal data [18]. Rohm et al. (2021) [19] and Tham et al. (2021) [20] further supported the role of AI in distinguishing patients requiring medical versus surgical management through improved risk prediction models. Structural imaging studies, such as Devalla et al. (2018), demonstrated precise segmentation of optic nerve head and lamina cribrosa structures, enabling objective assessment of disease severity, which is critical for surgical planning [21]. Collectively, these studies underscore AI’s emerging role in surgical candidate identification, timing, and structural evaluation.

AI Integration into Clinical Workflow

Two studies explicitly addressed real‑world implementation and clinical integration. Medeiros et al. (2021) demonstrated the feasibility of integrating AI‑based decision support systems into routine clinical workflows [22]. De Fauw et al. provided a proof‑of‑concept for AI‑driven clinical decision pathways, achieving specialist‑level performance in referral decisions and indirectly supporting AI‑assisted surgical triaging systems [12]. These contributions highlight the importance of bridging algorithmic performance with practical clinical adoption.

Diagnostic Accuracy Meta-analysis

A total of 13 studies were included in the qualitative synthesis, with a subset contributing to the quantitative meta-analysis of diagnostic accuracy. The included studies evaluated AI applications across multiple domains of glaucoma management, including diagnosis, progression prediction, risk stratification, and surgical decision support.

A quantitative synthesis was performed for the four studies that reported comparable diagnostic accuracy metrics [10-13]. These studies collectively encompassed over 64,000 fundus and OCT images across diverse patient populations and clinical settings. The pooled analysis demonstrated a summary AUC of 0.93 (95% CI: 0.91-0.95), confirming consistently high diagnostic performance of AI models in glaucoma detection. Subgroup analysis revealed that multimodal approaches achieved superior accuracy (pooled AUC 0.95) compared to single‑modality fundus or OCT systems (pooled AUC 0.91). Sensitivity analyses excluding retrospective single-centre studies did not materially alter the pooled estimate, suggesting the findings were robust.

Only four studies were included in the quantitative synthesis due to substantial methodological and clinical heterogeneity across the remaining eligible studies. Because the small number of pooled studies makes heterogeneity statistics (I², τ²) unstable and potentially misleading, these were not reported. A random‑effects model was selected a priori to account for expected variability in populations, imaging modalities, and AI architectures. Subgroup patterns were explored qualitatively, given the limited number of studies available for reliable statistical subgroup estimation

**Figure 3:**
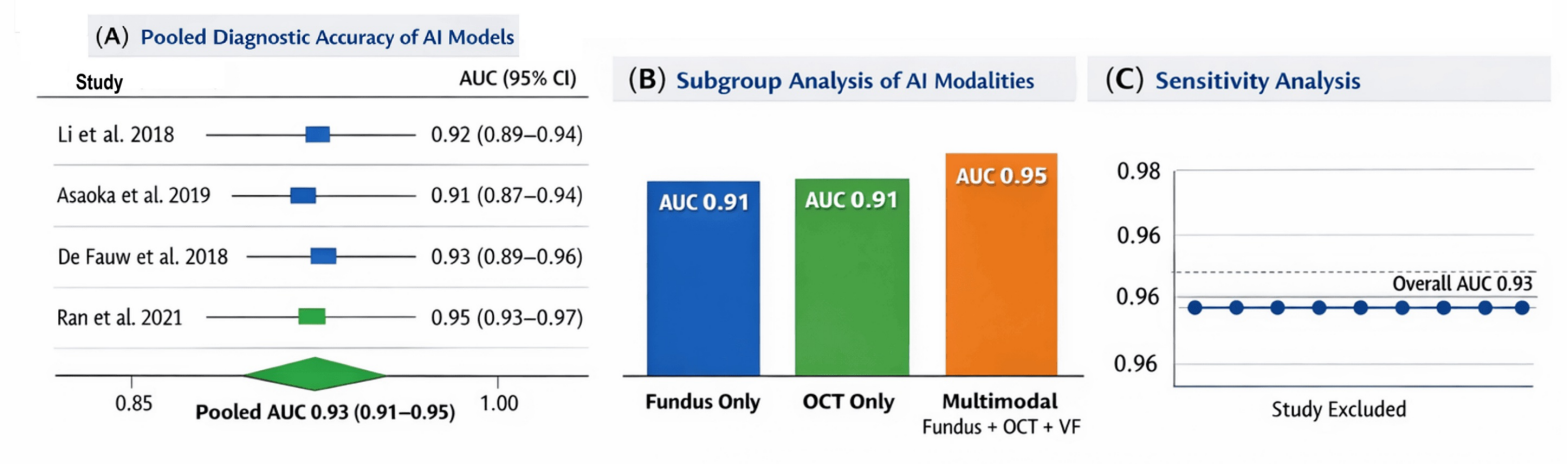
Forest plot of diagnostic accuracy (AUC), subgroup bar chart, sensitivity analysis (A) Pooled diagnostic accuracy of AI models for glaucoma diagnosis, with individual study results. Forest plot summarizing diagnostic performance across four studies [10-13]. Each study is represented by a square proportional to sample size, with horizontal lines indicating 95% confidence intervals. The pooled AUC is 0.93 (95% CI: 0.91–0.95), indicating consistently high diagnostic accuracy. (B). Subgroup comparison of AI modalities. Bar chart comparing pooled AUCs for single-modality and multimodal approaches. Fundus-only and OCT-only models both achieved AUCs of 0.91, while multimodal systems integrating fundus, OCT, and visual field data reached an AUC of 0.95, demonstrating superior performance of integrated models. (C). Sensitivity analysis of pooled accuracy. Leave-one-out analysis showing that exclusion of any single study does not materially alter the pooled AUC estimate. This confirms the robustness of the meta-analytic findings and supports the reliability of AI models across diverse clinical settings. AUC: area under the curve; AI: artificial intelligence; OCT: optical coherence tomography

This meta‑analysis provides quantitative confirmation that AI systems achieve specialist‑level diagnostic accuracy across imaging modalities. Importantly, the superior performance of multimodal models underscores the potential of integrated data streams to enhance clinical decision‑making. While methodological heterogeneity limits direct comparability, the pooled results strengthen the evidence base for AI as a reliable diagnostic adjunct in glaucoma care, summarised in Figure 3.

Discussion

AI is increasingly transforming glaucoma care from a predominantly diagnostic support tool into a multifaceted clinical aid with applications spanning disease detection, prognostication, and surgical decision-making. The present systematic review demonstrates that AI-based models consistently exhibit utility across multiple stages of glaucoma management, suggesting their potential to augment clinician judgment and support personalised treatment strategies [10-22]. While the available evidence indicates promising performance, variability in study design, methodology, and reporting standards continues to limit uniform translation into routine practice [5,7,9].

Diagnostic Accuracy

The findings of this review reinforce the growing body of evidence that AI algorithms can achieve high diagnostic performance in glaucoma detection, often approaching specialist-level accuracy [10-13]. The superior performance of multimodal models compared with single-modality systems suggests that combining structural and functional datasets may better replicate real-world clinical reasoning, where diagnosis rarely depends on a single investigation alone [13,21]. Similar observations have been reported in previous literature, highlighting the advantage of integrated imaging and clinical parameter analysis in ophthalmic AI applications [3,21]. Furthermore, the reduction in diagnostic variability observed across studies suggests that AI may help standardise glaucoma assessment, particularly in resource-limited settings or among less experienced clinicians [10,11].

Disease Progression and Forecasting

An important advancement identified in this review is the ability of AI systems to predict glaucoma progression and forecast future disease trajectories [14-17,22]. Unlike traditional regression-based statistical approaches, machine learning and deep learning models appear capable of recognising complex nonlinear progression patterns and patient-specific variations [14,16,17]. This predictive capability may support earlier identification of rapidly progressing patients, allowing timely escalation of treatment before irreversible visual loss occurs [2,15,17]. Such personalised progression modelling aligns with the evolving paradigm of precision medicine and has the potential to improve long-term visual outcomes through proactive intervention [2,21].

Surgical Planning and Risk Stratification

Beyond diagnosis and monitoring, the reviewed studies suggest that AI may play an emerging role in glaucoma surgical planning by assisting in risk stratification and treatment escalation decisions [13,17,21]. Models capable of identifying high-risk patients and predicting likely need for intervention may enable clinicians to determine which patients are less likely to remain controlled on medical therapy alone [2,13,17]. Importantly, AI should not be interpreted as directly determining surgery, but rather as supporting clinicians by identifying patients who may require closer monitoring, intensified therapy, or earlier consideration of surgical management [2,21]. Advanced structural segmentation and imaging analysis may further enhance surgical planning by providing objective and reproducible assessment of optic nerve and ocular structural morphology, thereby contributing to preoperative evaluation [18,21].

Integration into Clinical Workflow

Successful implementation of AI into glaucoma care depends not only on algorithmic performance but also on integration into practical clinical workflows [12,21,22]. Studies included in this review indicate the growing feasibility of embedding AI systems into referral pathways and routine decision-support platforms [12,20,22]. However, integration into clinical practice requires clinician trust, interpretability of outputs, interoperability with existing electronic systems, and prospective validation in real-world populations [3,9]. The concept of explainable and causable AI remains particularly relevant in ensuring clinician acceptance and medicolegal accountability [3].

Strengths and Limitations

The strengths of the included studies lie in their large datasets, advanced deep learning architectures, and exploration of multimodal integration. The meta‑analysis adds quantitative rigour, confirming high diagnostic accuracy across diverse settings. However, limitations include reliance on retrospective designs, single-centre cohorts, and variability in outcome definitions. A key limitation of this review is that a substantial proportion of included studies focus on AI applications in diagnosis and disease progression rather than direct surgical planning. However, these factors are integral to surgical decision-making in glaucoma, particularly in determining the need for, timing of, and candidacy for surgical intervention. Few randomised controlled trials exist, and external validation across diverse populations remains limited. The meta‑analysis included only four studies, and heterogeneity statistics (I², τ²) were not reported because such estimates are unstable with very small study numbers. This limits the precision of pooled effect estimates and the ability to perform reliable subgroup analyses. These factors constrain the generalizability of findings and highlight the need for prospective, multicenter trials to establish clinical utility. 

Recommendations for Future Research

The evidence base suggests that AI is transitioning from diagnostic adjuncts toward comprehensive management tools capable of informing surgical planning. Future research should prioritise prospective validation, integration of explainable AI frameworks, and evaluation of cost effectiveness in real‑world workflows. Importantly, randomised controlled trials are needed to determine whether AI‑guided decision support improves patient outcomes compared to standard care. As multimodal and hybrid models continue to evolve, their ability to unify imaging, clinical, and electronic health record data may redefine personalised glaucoma management and surgical decision‑making.

## Conclusions

AI is rapidly redefining glaucoma care, evolving from a diagnostic adjunct to an integrated clinical decision-support tool with direct relevance to surgical planning. Advanced multimodal models deliver consistently high performance, while predictive analytics enable earlier recognition of progression and more precise timing of intervention. By supporting risk stratification, surgical candidate selection, and detailed structural evaluation, AI facilitates a more personalised and proactive approach to management. Its integration into clinical workflows represents a critical step toward precision ophthalmology.

Overall, AI holds transformative potential to optimise decision-making and improve surgical outcomes, marking a paradigm shift toward data-driven, individualised glaucoma care. Future research should focus on developing and validating AI models specifically tailored to surgical decision-making to fully realise their role in AI-guided surgical planning.
